# Bacterial taxa decoupling with ageing

**DOI:** 10.18632/aging.104031

**Published:** 2020-08-28

**Authors:** Andrés Moya, Susana Ruiz-Ruiz, Manuel Ferrer

**Affiliations:** 1 Institute of Integrative Systems Biology (I2Sysbio), University of València and Spanish National Research Council (CSIC), València, Spain; 2 Foundation for the Promotion of Sanitary and Biomedical Research of Valencian Community (FISABIO), València, Spain; 3 CIBER in Epidemiology and Public Health, Madrid, Spain; 4 Institute of Catalysis, Spanish National Research Council (CSIC), Madrid, Spain

**Keywords:** ageing, indole, tryptophan

Ageing-associate physiological changes are also reflected in the gut microbiota, in such a way that taxa and associated functions differ between younger an older people [1]. Moreover, we have compelling evidence that some gut bacterial functions are increasingly decoupled with age, contradicting the general assumption that the gut microbiota as a whole systematically contributes to our health status as we age. By performing shotgun and target proteomics and target metabolomics studies, Ruiz et al. [2] have identified a reproducible microbiome profile that differed among age groups (i.e., infants, adults and elderly) but, more importantly, they detected metabolic deficits in our gut bacteria as we get older [2]. The shotgun proteomes revealed that the number of proteins produced by our gut bacteria increases with age and that many of them differ from those harbored when younger. By using target proteomics these differences were found to be statistically significant in two of the 437 protein-functions identified, namely KO1667 (TnaA, tryptophanase) and KO1696 (TrpB, tryptophan synthase), given that their expression values were high in infants, intermediate in adults and practically null in the elderly. TrpB is essential for tryptophan synthesis and TnaA is essential for converting tryptophan into indole. The progressive decline in the intestinal microbiota from childhood to old age of these two essential products, tryptophan and indole, were validated by measuring their relative concentration in the fecal fluid using a target metabolomics approach, observing a spectacular drop in both of them with age. Half-life analyses showed that these molecules are reduced to 50% between 11-31 years with an even greater reduction of 90% between 34-54 years of age. In a previous study by our group [3] we also detected a low presence of *tna*A and *trp*B genes in the gut microbiota of the adult population. We therefore speculate, from an evolutionary perspective, that those genes and the corresponding bacterial taxa could be maximized in the reproductive period of the human species.

Sonowal et al. [4] used animal models (*Caenorhabditis elegans*, *Drosophila melanogaster* and mouse) to demonstrate the relevance that tryptophan and indole have in what they call the “quality of life” in old age, because their absence was linked to frailty syndromes [4]. The aforementioned authors measured traits such as mandibular movement, locomotion, movement of wings, etc., in old individuals of these species and demonstrated that when the molecules were supplied directly or were provided by microorganisms capable of synthesizing them, the individuals enjoyed better quality of life than when provision failed. Apparently, the lack of these molecules made the elderly organisms fragile.

If tryptophan and indole contribute to preventing fragility, healthy elderly subjects may possibly obtain them by other sources, whereas elderly people diagnosed with frailty syndrome [5] have low levels of these circulating molecules because those sources are lacking. According to our results, we think that both healthy and fragile elderly have a lack of bacterial taxa responsible for producing tryptophan and indole or there is some inhibitory action on those taxa if they are present. On the other hand, this supports the hypothesis of a functional decoupling between gut microbiota and the human host at older ages, contradicting the notion that gut microbes always benefit their host’s health status. From an evolutionary biology perspective, if we consider that gut microbes and their hosts are evolving together, albeit not necessarily as a unit of evolution, then we can expect periods during host life, particularly at reproductive ages, when coupling is higher than in other periods. On the other hand, we should also expect adaptive host responses to solve gut bacterial decoupling [6,7]. A better understanding of the processes through which the microbiota is coupled and decoupled from its host, and the reasons and when they occur, will contribute to establishing a more solid theoretical framework for the development of gut microbiota modulation strategies in the many contexts in which our resident microbes affect our health. These considerations, on the other hand, could have relevant clinical implications insofar as they will provide clues about specific dietary recommendations, particularly the repercussions of frailty in old age on the life quality of many people, and the costs they represent for the health system.
